# Clinical Outcome and Fusion Rates after the First 30 Extreme Lateral Interbody Fusions

**DOI:** 10.1100/2012/246989

**Published:** 2012-11-01

**Authors:** Gregory M. Malham, Ngaire J. Ellis, Rhiannon M. Parker, Kevin A. Seex

**Affiliations:** ^1^Neuroscience Institute, Epworth Hospital, Bridge Road, Melbourne, VIC 3121, Australia; ^2^Greg Malham Neurosurgeon, Suite 2, Level 1, 517 St. Kilda Road, Melbourne, VIC 3004, Australia; ^3^Department of Neurosurgery, Macquarie University, Sydney, NSW 2109, Australia

## Abstract

*Introduction*. The lateral transpsoas approach for lumbar interbody fusion (XLIF) is gaining popularity. Studies examining a surgeon's early experience are rare. We aim to report treatment, complication, clinical, and radiographic outcomes in an early series of patients. *Methods*. Prospective data from the first thirty patients treated with XLIF by a single surgeon was reviewed. Outcome measures included pain, disability, and quality of life assessment. Radiographic assessment of fusion was performed by computed tomography. *Results*. Average follow-up was 11.5 months, operative time was 60 minutes per level and blood loss was 50 mL. Complications were observed: clinical subsidence, cage breakage upon insertion, new postoperative motor deficit and bowel injury. Approach side-effects were radiographic subsidence and anterior thigh sensory changes. Two patients required reoperation; microforaminotomy and pedicle screw fixation respectively. VAS back and leg pain decreased 63% and 56%, respectively. ODI improved 41.2% with 51.3% and 8.1% improvements in PCS and MCS. Complete fusion (last follow-up) was observed in 85%. *Conclusion*. The XLIF approach provides superior treatment, clinical outcomes and fusion rates compared to conventional surgical approaches with lowered complication rates. Mentor supervision for early cases and strict adherence to the surgical technique including neuromonitoring is essential.

## 1. Introduction

The lateral transpsoas approach for anterior lumbar interbody fusion (extreme lateral interbody fusion (XLIF)) was developed as a less-invasive alternative to conventional anterior and posterior approaches for interbody fusion [1]. Similar to anterior exposures for lumbar interbody fusion, the lateral approach allows for placement of a wide footprint intervertebral cage with wide apertures to provide superior anterior column realignment [2, 3] as well as a healthy fusion environment [4], without anterior and posterior longitudinal ligament (ALL and PLL) resection. In addition, the lateral approach mitigates many of the risks more common to traditional approaches, namely, vascular and visceral risks associated with anterior approaches [5–8] and the neural complications and bony resection common to posterior approaches [9, 10]. However, safe passage through the psoas muscle requires neuromonitoring to identify the nerves of the lumbar plexus, the injury of which represents a significant risk of the approach. 

 Since the introduction of the approach in the literature in 2006 [1], the procedure has increased in popularity, and reports of safety and outcome continue to be needed to fully validate the approach, especially during early cases of a new approach where a learning curve may be present [11, 12]. The purpose of this study was to examine clinical and radiographic outcomes in the first thirty patients treated with the XLIF approach by one surgeon in Melbourne, Australia. 

## 2. Materials and Methods

Data were collected through a prospective registry, with retrospective analysis performed of the first 30 (consecutive) patients treated with extreme lateral interbody fusion (XLIF, NuVasive Inc., San Diego, CA, USA) by a single surgeon (GM) in Melbourne, Australia from February 2011 to October 2011. Patients were treated only after failure of extended conservative therapy and imaging studies, including dynamic (flexion, extension, and lateral bending) radiography, computed tomography (CT) coregistered with bone scans, magnetic resonance imaging (MRI), and bone mineral density (DEXA) scans, as appropriate. Data were collected preoperatively and then postoperatively at standard follow-up intervals for one year postoperatively. 

Baseline patient information included basic demographic information as well as the primary indication for surgery and baseline medical comorbidities. Treatment information included levels treated, biologics and fixation used, and the presence of any procedural side effects, complications, or reoperations. Patient-reported outcomes included minimum, maximum, and average back and leg pain (LBP and LP) (visual analogue scale (VAS)), disability (Oswestry Disability Index (ODI)) and quality of life (SF-36 physical and mental component scores (PCS and MCS)). Fusion was assessed using high definition (HD) CT (Somatom scanner) taken one to two days postoperatively to assess instrumentation placement and then between six and twelve months postoperatively to assess fusion status. Fusion was defined as the presence of bridging interbody trabecular bone [13] and was determined by a third-party radiologist from within the treating institution.

The surgical procedure has previously been described [1] but involves a 90° off-midline retroperitoneal approach to the anterior lumbar spine with blunt dissection through the fibres of the psoas muscle to the lateral border of the disc space. Passage through the psoas muscle, avoiding the nerves of the lumbar plexus, is accomplished using a neuromonitoring system (NV JJB/M5, NuVasive, Inc.) integrated into approach and procedural instrumentation. Neuromonitoring with this system provides real-time and surgeon-directed discrete-threshold electromyographic responses to provide geographic information about the presence of motor nerves relative to procedural instrumentation [14, 15]. One thoracic level was treated (T6-7), and a similar procedure to the lumbar XLIF procedure was followed, though using a transpleural lateral approach, as has also been previously described [16, 17]. Direct decompressions were performed when required. 

All patients were fitted with intervertebral polyetheretherketone (PEEK) cage(s) (CoRoent, NuVasive, Inc.) filled with a combination of bone morphogenetic protein (rhBMP-2 (BMP), Infuse, Medtronic, Inc., Memphis, TN, USA) and Mastergraft *β*-TCP granules (Medtronic, Inc.). BMP has a fixed concentration of 1.5 mg/cc, and the dose used per level was volume dependent (i.e., the internal volume of cage equalled BMP volume in cc), using (a small kit of BMP (2.8 cc providing a 4.2 mg dose), per the manufacturers recommendation, following a one-hour absorption into the carrier period. No BMP was placed outside the cage. Supplemental internal fixation was applied as needed. 

Statistical analyses included frequency testing for demographic and treatment variables, paired *t*-tests comparing clinical outcomes from preoperative levels, and fisher exact tests for comparisons of the frequency of events between groups. Statistical analysis was carried out using SPSS v. 19.0 (SPSS IBM, Chicago, IL, USA) with statistical significance measured at *P* < 0.05.

## 3. Results

The first thirty (30) patients treated with XLIF were included in the analysis and had a mean age of 63 years with a mean body mass index (BMI) of 26.7, and 20 (67%) were female. Baseline comorbidities included tobacco use (20%), diabetes mellitus (13%), and prior lumbar spine surgery (20%). The most common primary diagnoses included degenerative disc disease (41%), spondylolisthesis (31%), and degenerative scoliosis (24%). In 30 patients, 43 levels (1.4 per patient, range 1–3) were treated with the most common levels being L3-4 and L4-5 (in 57% of patients, each). Supplemental internal fixation was used in 15 (50%) patients and included pedicle screw fixation in 13 and interspinous plating in two patients. Staging of secondary procedures (decompressions and/or fixation) occurred in 47% of cases. A summary of baseline and treatment information is included in Table 1.

Average operating time per level was 60 minutes with a mean blood loss of 50 mL per level (range 10–150 mL).

**Figure 1:**
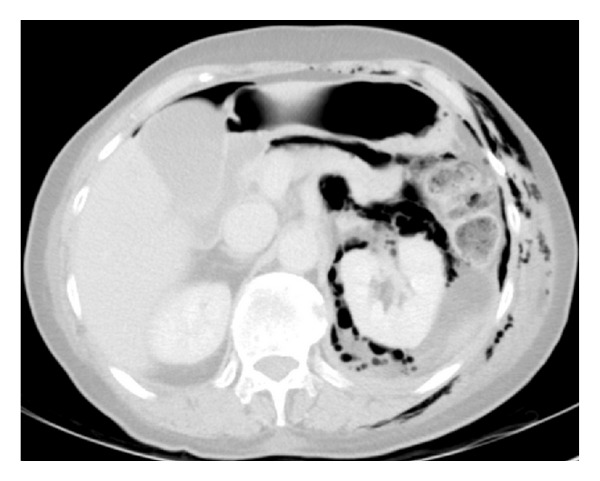
Abdominal axial computed tomography (CT) showing intraperitoneal free air following unrecognized bowel perforation.

Four (13%) complications were observed. One large bowel injury occurred in a thin 53-year-old female patient who underwent a left-sided approach for a L3-5 XLIF with posterior instrumentation for disabling low back pain above a previous L5-S1 fusion. The patient had a past history of midline laparotomy for bowel obstruction performed 20 years previously. On day three postoperatively the patient developed left lower quadrant abdominal pain with tenderness and tachypnoea. Chest and abdominal plain radiographs were indeterminate for free air, but abdominal CT demonstrated intraperitoneal air (Figure 1). Urgent laparotomy found that the descending colon had been perforated adjacent to the L4-5 level on the side ipsilateral to the approach. One patient developed a new motor deficit immediately evident postoperatively with 4/5 power quadriceps due to a posteriorly placed cage which resulted in a L2 radiculopathy that partially resolved with persistent 4+/5 weakness at 12 months. One instance of symptomatic subsidence was observed in the form of unilateral disc space collapse with a 22 mm-wide cage inferior to a prior fusion, and while a reoperation was not required, fusion was not evident at 12 months. Finally, there was one instance of cage breakage following an attempted forceful impaction of an 8 mm cage into a collapsed L3-4 disc space. In addition, three cases of asymptomatic (radiographic) subsidence (<25% height loss) were observed without sequelae. Of the four instances of cage subsidence, three included 18 mm cages (two standalone, one bilateral pedicle fixation) and one with 22 mm (standalone).

Side effects of the approach were observed, with five cases of anterior thigh sensory changes (dysesthesias), four of which had resolved by six weeks postoperative and one of which was persistent at last followup (12 months). Of these, three occurred within the first 10, and none occurred in the last 10, patients of the series. Complications and side effects are included in Table 3. 

Two patients required reoperation: one underwent a microforaminotomy for a posteriorly placed cage and a second underwent bilateral pedicle fixation for symptomatic facet arthropathy. 

Four patients were lost to followup. All patients or their representatives were contacted by phone for followup, and reasons for noncompliance included one who is a workers compensation case and refused followup, another is an elderly women who was satisfied with her outcome but was unable to travel to the office, and another whose son reported that the patient had become morbidly obese (130 kg) and was now agoraphobic and unable to leave the house. One patient was unable to be contacted. 

**Figure 2:**
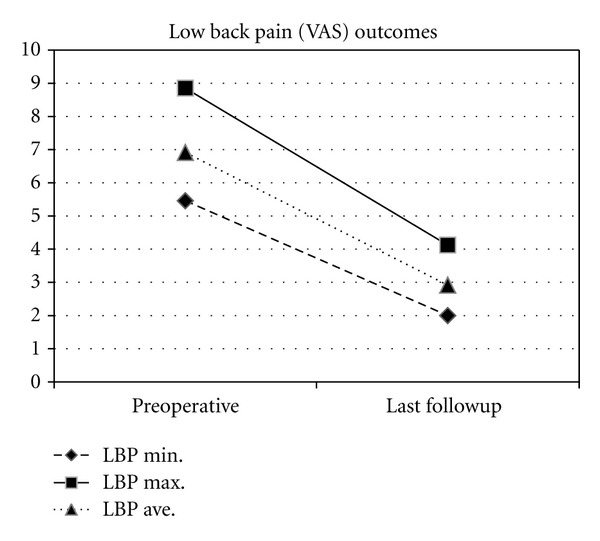
Change in minimum, maximum, and average low back pain (LBP) from preoperative to last followup (mean 11.5 months).

**Figure 3:**
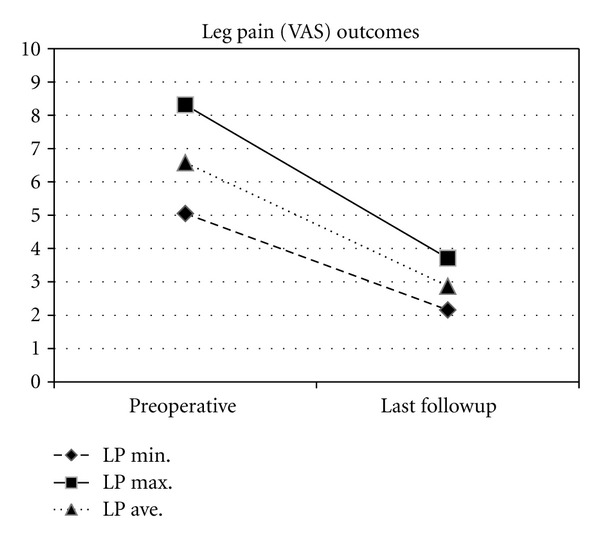
Change in minimum, maximum, and average leg pain (LP) from preoperative to last followup (mean 11.5 months).

**Figure 4:**
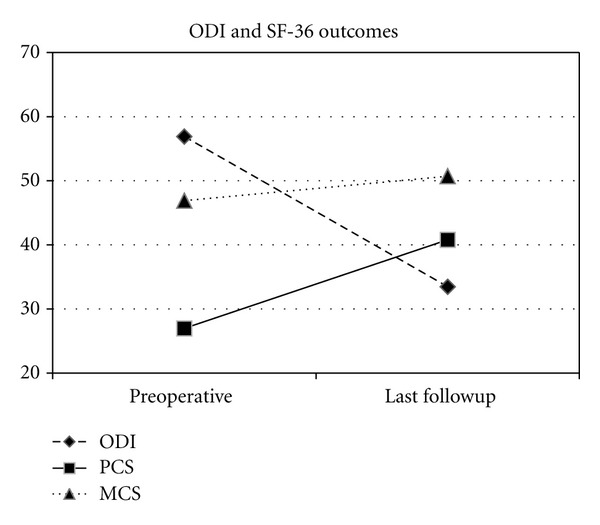
Change in average disability (ODI), and physical and mental quality of life (PCS and MCS) from preoperative to last followup (mean 11.5 months).

**Figure 5:**
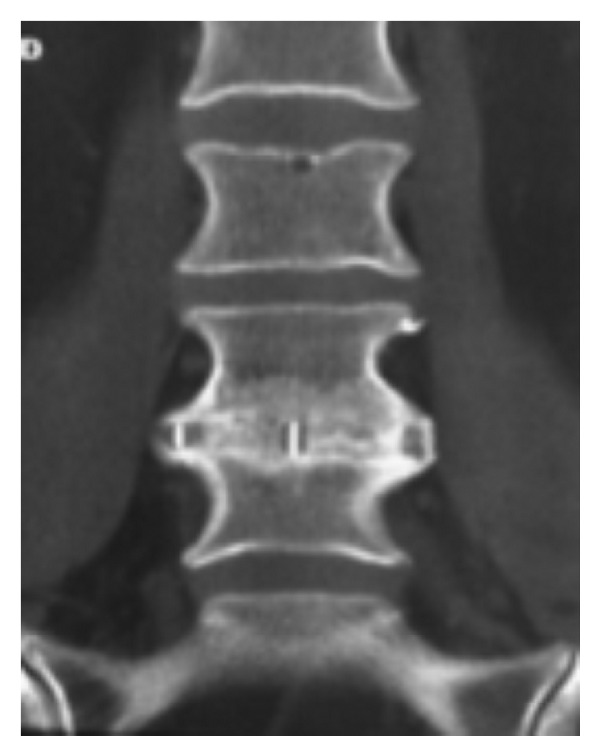
Coronal computed tomography (CT) showing solid arthrodesis at 12 months postoperative following L4-5 XLIF.

 Of those able to be followed (26), average followup was 11.5 months (range 9–12). Average back and leg pain (in those with leg pain) improved 6.9 and 6.6 to 2.9 and 2.9, representing a 63% and 56% improvement, respectively (Figures 2 and 3). Disability (ODI) improved from 56.9 preoperatively to 33.5 at last followup (41.2%) with PCS and MCS improving 51.3% (27.0 to 40.8) and 8.1% (46.9 to 50.7), respectively (Figure 4). All clinical results were statistically significantly improved from baseline (*P* < 0.001) except for MCS (*P* = 0.200). Fusion rate confirmed on HD CT coronal views (Figure 5) progressed from 46% (12/26) at 6 months to 58% (15/26) at 9 months and 85% (22/26) at 12 months postoperatively (Table 2). In patients with supplemental internal fixation, a 92% (12/13) fusion rate was observed, while without fixation only 77% (10/13) of patients exhibited complete fusion at 12 months, a difference which was not statistically significant (*P* = 0.593).

## 4. Discussion

The primary indications for the XLIF procedure are thoracolumbar pathology from approximately T4 through L5 (limited superiorly by the axilla and interiorly by the iliac crest) and include symptomatic disc degeneration [18], degenerative scoliosis [19, 20], spondylolisthesis, adjacent segment disease [21], as well as traumatic, tumor, and infection pathologies [22–24]. Relative contraindications for XLIF included L5-S1 pathology, retroperitoneal adhesions, and early bifurcation of the iliac vessels. Preoperative assessment of the neurovascular complex at each level to be treated on axial MRI is essential to have a preoperative understanding of regional anatomy as it relates to the lateral approach [25]. 

 In the first 30 cases of XLIF at one institution, the authors observed a 13% complication rate in 30 patients with two reoperations occurring. Mean followup was 11.5 months and low back and leg pain decreased by 63% and 56%, respectively, with similar improvements in disability (41.2%) and physical and mental quality of life (51.3% & 8.1%, resp.). 

 In comparison with alternative approaches for lumbar interbody fusion, complications rates with transforaminal and posterior lumbar interbody fusion (T/PLIF) have generally been reported in elevated ranges compared to the current series. In 2009, Rihn et al. [9] reported on a series of 119 TLIF cases performed at Thomas Jefferson University Hospital. An overall complication rate of 46% (55) was observed in 35% (40) of patients. While 10 complications were attributed to iliac crest bone graft harvesting, there was a 10.9% rate of new postoperative radiculitis, a 5% infection rate, and a 10.1% reoperation rate. Similarly, Okuda et al. [26] in 2006 reported the surgical complications of 251 PLIF patients treated at a single institution. In this series, the authors found an intraoperative complication rate of 10.3% with a new postoperative neurologic deficit rate of 8.3% (21; 19 motor, 2 sensory), with 32% of those classified as slight, 47% severe, and 21% permanent. Results in the current series, having observed a 13% complication rate, is favorable to these similar study-design historical results, even when factoring in that cases in the current series represented the adoption of a new procedure [11, 12]. In total, six (20%) neural adverse events occurred, one motor complication and 5 sensory side-effects, rates which are consistent with high-quality prospective multicenter studies of XLIF performed using surgeons already familiar with the procedure [14]. Tohmeh et al. [14] observed a 17.5% rate of transient anterior thigh sensory changes postoperatively with a 2.9% new motor deficit rate in 102 XLIF patients treated at L3-4 and/or L4-5. In addition, the single incidence of motor injury occurred as a result of a misplaced cage (case 6) rather than during direct injury by procedural instrumentation during the approach for procedure. When considering the generally transient nature of the expected sensory nerve irritation, the incidence of neural events (the most apparent anatomical risk during the procedure) also compares favorably to posterior approaches. 

Anatomically, the sensory nerves at risk with this operation are the ilioinguinal, iliohypogastric, lateral femoral cutaneous, and genitofemoral nerve [27]. The first three nerves are at risk of injury in the approach to the psoas. The genitofemoral nerve arises from the L1 and L2 nerve roots, traverses the psoas, and descends along the anteromedial border of the psoas deep to its fascia [28]. The nerve crosses the L2-3 disc space and may be injured anywhere along its course [28, 29] though the risk is somewhat mitigated by more posterior docking on the lateral aspect of the disc space, enabled by neuromonitoring of the more-posterior motor nerves of the lumbar plexus [15]. The patients in this series that experienced the side effect of genitofemoral irritation, which are relatively common with this procedure, usually resolve within 6 weeks, but persistence has been reported [14, 30] as in one of the five cases in this series. In the current series, we observed a reduction in the incidence of sensory side effects from early cases (20% rate in the first 20 cases) compared to later (0% in last 10 cases) though the difference in rate was not statistically significant (*P* = 0.140). Potential reasons for the decrease in these events may include decreased duration of time and the psoas muscle was under retraction (procedural efficiency) and increased comfort with more posterior docking (avoiding the more anterior genitofemoral nerve) with incremental adherence to neuromonitoring. 

Radiographic subsidence was observed in three cases, with one instance of both radiographic and clinical subsidence. Factors thought to contribute to cage subsidence are the narrower 18 mm cages, osteoporosis, the use of BMP-2, the use of standalone cages, and iatrogenic endplate violation [31, 32]. Three of the four cage subsidence in this series occurred with 18 mm standalone cages. The symptomatic subsidence occurred six weeks postoperatively after the insertion of a 22 mm standalone cage packed with BMP-2 inferior to a previous fusion in a patient with normal bone density. This may reflect increased biomechanical stress at the L4-5 level as well as the osteolytic, inflammatory phase of BMP-2 [32]. 

In the patient who experienced the unrecognized bowel injury, the injury likely occurred during placement of the initial dilator, which was delivered at an angle from the plane perpendicular to the floor, in a deviation from the prescribed surgical technique. The patient required a Hartmann's colostomy that was reversed two months later. She recovered without infection and reported significant improvement in low back pain and mobility. Bowel injury following XLIF has previously been reported as a complication of the approach, both acute and delayed [33]. 

Clinical and radiographic outcomes were consistent with previously-reported results which showed fusion rate ranges between 91% and 100% (though generally with more extended followup), 37% to 80% reduction in low back pain, and a 39% to 82.1% reduction in disability (ODI) [34]. These results are similar or superior to conventional surgical approaches. Blumenthal et al. [35], as a part of the Charité artificial lumbar disc Food and Drug Administration (FDA) investigation, reported a 47.6% improvement in low back pain at 24 months postoperative in the ALIF fusion control group with a 41.5% improvement in ODI. Similar results were seen in ALIF by Kuslich et al. [36] in 1998, showing an improvement of 42% in pain and 31.5% in disability at 24 months postoperatively.

In the current series, the relatively lower early fusion rate seen in standalone cases may suggest an extended healing period due to the less-rigid segmental environment to promote fusion [37]. While this has yet to be formally studied, several studies of standalone XLIF show that some consistency with this notion though, also of note, is that progression to complete fusion does generally occur [38–41]. 

## 5. Conclusion

In summary, these data represent generally superior treatment (blood loss and operative time), clinical (pain, disability, and quality of life), and fusion rates using the XLIF approach compared to conventional surgical approaches with substantially lowered complication rates. With specific training, mentor supervision for early cases, and strict adherence to surgical technique including neuromonitoring, surgeons can anticipate low perioperative morbidity even in the early period following the adoption of the approach.

## Figures and Tables

**Table 1:** Listing of patient demographic and treatment information.

Characteristic	Statistic *n* = 30
Mean age in years (stdev) (range)	62.7 (10.5) (30–81)
Female (%)	20 (66.7)
Mean body mass index (BMI), (stdev) (range)	26.7 (5.4) (17.6–37.9)
Comorbidities	
Comorbidity type	
Tobacco use (%)	6 (20)
Diabetes (%)	4 (13)
Any prior lumbar spine surgery (%)	6 (20)
Lami/MLD (%)	4 (67)
Fusion (%)	2 (33)
Primary diagnosis	*n* = 29 (1 missing)
Degenerative disc disease (%)	12 (41)
Herniated nucleus pulposus (%)	1 (3)
Spondylolisthesis (%)	9 (31)
Scoliosis (%)	7 (24)
Levels treated (mean per patient) (range)	43 (1.4) (1–3)
T6-7 (% of levels) (% of patients)	1 (2) (3)
L1-L2 (% of levels) (% of patients)	1 (2) (3)
L2-L3 (% of levels) (% of patients)	6 (14) (20)
L3-L4 (% of levels) (% of patients)	17 (40) (57)
L4-L5 (% of levels) (% of patients)	17 (40) (57)
Biologics used	
rhBMP-2 (%)	30 (100)
Fixation type (%)	
Interspinous plating (%)	2 (7)
Transpedicular fixation (%)	13 (40)
Unilateral (%)	2 (15)
Bilateral (%)	11 (85)
Standalone (%)	15 (50)
Staged fixation?	
Yes (%)	14 (47)
No (%)	16 (53)

*n*: number of patients; stdev: standard deviation; Lami: laminectomy; MLD: microlumbar discectomy.

**Table 2:** XLIF fusion rates.

Time postoperatively	Fusion rate
6 months	46% (12/26)
9 months	58% (15/26)
12 months	85% (22/26)

**Table 3:** Complications and side effects.

Patient number	Levels (mean)	Dysaesthesia	Motor deficit	Reoperation	Subsidence	Cage breakage	Bowel injury
1–10	1.1	3	1	1	0	0	0
11–20	1.3	2	0	0	2	0	0
21–30	1.5	0	0	1	2	1	1

Totals	42	5	1	2	4	1	1
